# Photoperiod as a proximate factor in control of seasonality in the subtropical male Tree Sparrow, *Passer montanus*

**DOI:** 10.1186/1742-9994-8-1

**Published:** 2011-01-11

**Authors:** Anand S Dixit, Namram S Singh

**Affiliations:** 1 Department of Zoology, North-Eastern Hill University, Shillong-793022, Meghalaya, India

## Abstract

**Background:**

Most species of birds exhibit well-defined seasonality in their various physiological and behavioral functions like reproduction, molt, bill color etc. such that they occur at the most appropriate time of the year. Day length has been shown to be a major source of temporal information regulating seasonal reproduction and associated events in a number of avian species. The present study aims to investigate the role of photoperiod in control of seasonal cycles in the subtropical male tree sparrow (*Passer montanus*) and to compare its responses at Shillong (Latitude 25°34'N, Longitude 91°53'E) with those exhibited by its conspecifics and related species at other latitudes.

**Results:**

Initial experiment involving study of seasonal cycles revealed that the wild tree sparrows posses definite seasonal cycles of testicular volume, molt and bill color. These cycles were found remarkably linked to annual solar cycle suggesting the possibility of their photoperiodic control. To confirm this possibility in the next experiment, the photosensitive birds were exposed to three different light-dark regimes that were close to what they experience at this latitude: 9L/15D (close to shortest day length), 12L/12D (equinox day length) and 14L/10D (close to longest day length) for 18 months. Tree sparrows showed testicular growth followed by regression and development of photorefractoriness, molting and bill color changes only under long daily photoperiods (12 L and 14 L) but not under short daily photoperiod (9 L). Birds, under stimulatory photoperiods, did not show reinitiation of the above responses after the completion of initiation regression cycle even after their exposure to these photoperiods for 18 months. This precludes the possibility of circannual rhythm generation and suggests the involvement of photoperiodic mechanism in control of their seasonal cycles. Further, replacement of body and primary feathers progressed with gonadal regression only under long days suggesting that the two high energy demanding events of reproduction and molt are phased at two different times in the annual cycle of the bird and are photoperiodically regulated. Results of the final experiment involving exposure of photosensitive birds to a variety of photoperiodic treatments (9L/15D, 10L/14D, 11L/13D, 12L/12D, 14L/10D and 16L/8D) for 30 days suggested that the light falling for 11 h or more is important in inducing testicular growth and function in this species.

**Conclusion:**

These results clearly indicate that despite of small photofluctuation, subtropical tree sparrows are capable of fine discrimination of photoperiodic information and use day length as a proximate environmental factor to time their seasonal responses similar to their conspecifics and related species at other latitudes suggesting the conservation of photoperiodic control mechanism in them.

## Background

Most birds exhibit well-defined seasonality in their various physiological and behavioral functions including gonadal growth and development, molt, body mass, bill coloration, hormone levels, song production etc.[1-3]. Most of these functions generally centre on reproduction that occurs at the most appropriate time of the year when the food resources in the wild are optimally present and the chances of survival of the youngs and parents are maximum [4]. Therefore, the timing of actual reproduction is critical for the species. Because change in photoperiod is entirely predictable at given latitude, both within and between years, it is used as a reliable cue to time the physiological preparations for three major life-history stages: reproduction, molt and migration [5-7]. Day length has been shown as a major source of temporal information regulating seasonal responses in a number of avian species inhabiting both mid and high latitudes [2,8-11]. On the other hand, there is little evidence about nonphotic cues as *zeitgeber *in the synchronization of seasonal cycles [12,13]. These may act mainly as supplementary and modifying cues [14,15]. The role played by light in controlling reproduction and associated functions of temperate zone birds is relatively well established [16,17]. Less is known about the importance of day length in controlling these functions in tropical and subtropical birds. Because of small annual variations in the tropics and subtropics, photoperiod has been speculated to be of little use in regulating metabolic and reproductive functions of birds [18]. This possibility has been examined in some tropical and subtropical species and it has been found that in spite of small photofluctuation, light plays a much more significant role than has hitherto been assumed [19-21].

Birds tend to adapt to daily light-dark cycles (LD cycles) using their endogenous time-keeping device(s), called "clocks" because of their great precision in the timing of various behavioral and physiological events. Day length interacts with these clocks and induces seasonal responses. In general, seasonal cycles appear to be regulated by two mechanisms [22]: the photoperiodism and circannual rhythm generation. In photoperiodism, the environmental photoperiod (= daily light period) is involved in generation of seasonal rhythms through induction and termination of physiological processes. An endogenous clock enables the bird to identify the time when to switch on (photoinduction) and when to switch off (photorefractoriness) its physiological mechanisms so that seasonal events occur at the most suited time of the year. The coincidence of daily light with the period of maximum inducibility of the endogenous clock [23] occurring about 12 hours (12 h) after the sunrise in a long day breeder, as in spring and summer months, is read as a "long day" and leads to the photoperiodic induction. Failure of such coincidence, as would be the case during the winter months when daily light is < 12 h per day is read as a "short day"; consequently, there is no photoperiodic induction [23]. Instead, short days tend to mediate the recovery of the photosensitivity in refractory individuals [24]. The other mechanism is the circannual rhythm generation, in which a self-sustained endogenous rhythmicity of about a year times these component events as reported in few avian species [25,19]. Thus, as is true of circadian rhythms, circannual rhythms are both endogenously generated and synchronized by environmental factors such as day length (photoperiod), food availability etc.[25-27]. Endogenous circannual rhythms have been experimentally demonstrated in more than twenty migratory and resident bird species from both tropical and temperate regions in controlling reproduction, migration, molt and *zugunruhe *[28-30]. The role of photoperiod in such a temporal scheme is limited where it is used to synchronize the circannual rhythm to the calendar year, but it does not alter the overall temporal course of seasonal programming of the annual events. However, it is still not clear whether photoperiod acts directly to control physiology, or if it synchronizes an innate circannual rhythm of life cycle events and also whether circannual and circadian rhythmicities are completely separated from each other [4].

Reproduction and plumage molt are high energy demanding processes in the life cycle of birds [31-33]. Feather loss may impair flight performance [34] making molt and reproduction incompatible [35]. Accordingly, both the events are so timed that they do not overlap as molt breeding overlap is inherently costly [36]. The photoperiodic environment to which the birds are exposed to differs with the latitude and seasons. Therefore, a photoperiodic species may show latitude and season-dependent photoperiodic adaptations. There are examples where the birds of same latitude breed at different times in different habitats, and in the same habitat between different years [37,38]. Clearly, non photoperiodic cues may play a significant role in modulating the timing of photoperiodically induced gonadal maturation and breeding. The fact that both high and low latitude species are capable of very fine discrimination of even small photoperiodic changes reveals that they represent adaptation in inhabiting different photoperiodic environments. Thus, it is more interesting to study photoperiodic adaptations in the bird species having wide distribution covering different latitudes. Therefore, it is proposed to study the detailed pattern of seasonality in a subtropical population of tree sparrow, a resident bird, inhabiting North-East part of India (Shillong: Lat. 25°34'N, Long. 91°53'E; day length fluctuation: 3 h and 15 min.) and then to investigate whether annual variation of day length indeed plays a role in timing seasonal breeding and associated events in this species and finally to compare its seasonal responses with those of its conspecifics and other related species in the subtropical and temperate zones. In different experiments, we have (i) described annual seasonal cycles in the wild birds in relation to different environmental factors, (ii) investigated seasonal cycles under programmed photoperiodic schedules (9L/15D, corresponding to shortest day length; 12L/12D, equinox day length; and 14L/10D, corresponding to longest day length), and (iii) defined critical day length for testicular response.

## Results

### Experiment 1: seasonal cycles in the wild birds

**Figure 1:**
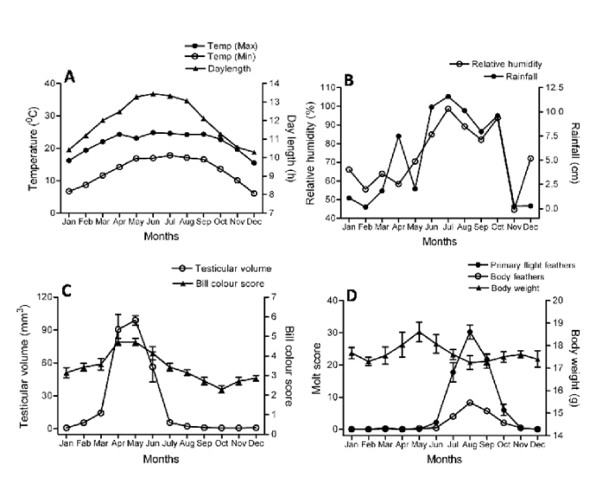
**Effects of ambient environmental factors on seasonal cycles of the male tree sparrow (*Passer montanus*)**. Annual changes in temperature, day length, relative humidity and rainfall are presented as means for the months. Birds (n = 7) were capture in the middle of every month and observations were recorded on the changes in testicular volume, bill color, body weight and molt of wing primary and body feathers. The data are presented in means ± s.e.m. Though a slight increase in testicular volume was observed during February to March, a significant testicular growth was noticed only in the month of April with the increasing day lengths in spring. Molting of wing primary and body feathers progressed with gonadal regression showing definite annual cyclicity. No significant changes in body weight were observed throughout the study. The birds posses distinct annual cycles of testicular growth and bill color that showed close correspondence with the annual day length cycle.

Results are shown in figures 1A, B, C and 1D. One-way ANOVA revealed significant changes in testicular volume (F_11,72 _= 40.84, *P *< 0.0001), bill color (F_11,72 _= 12.84, *P *< 0.0001), molting in wing primaries (F_11,72 _= 68.35, *P *< 0.0001) and body feathers (F_11,72 _= 59.62, *P *< 0.0001) over the year. Annual testicular cycle in the wild sparrows follows seasonal changes in day length at shillong. In this study, a small increase in testicular volume was noticed in February and March but a significant increase was observed only in the month of April that reached to its peak in May (mean TV = 98.9 ± 4.3 mm^3^). Birds showed testicular regression in June that progressed through July reaching to its minimum value in September (mean TV = 1.1 ± 0.1 mm^3^) which was maintained till December. Monthly changes in bill color ran parallel to annual testicular cycle in sparrows showing maximum scores in the months of April and May (mean bill coloration score = 4.71 ± 0.18) and minimum in October (mean bill coloration score = 2.28 ± 0.18). Molting in the wing primaries and body feathers progressed with gonadal regression extending from June to November with peak in the month of August (mean wing primaries score = 30.28 ± 2.15 and mean body feather score = 8.28 ± 0.78). One-way ANOVA revealed no significant difference in the body weight (F_11, 72 _= 1.451, *P *= 0.1696) of sparrows throughout the year during which the mean monthly body weight varied only from 17.3-18.6 g.

### Experiment 2: seasonal cycles under programmed schedules

**Figure 2:**
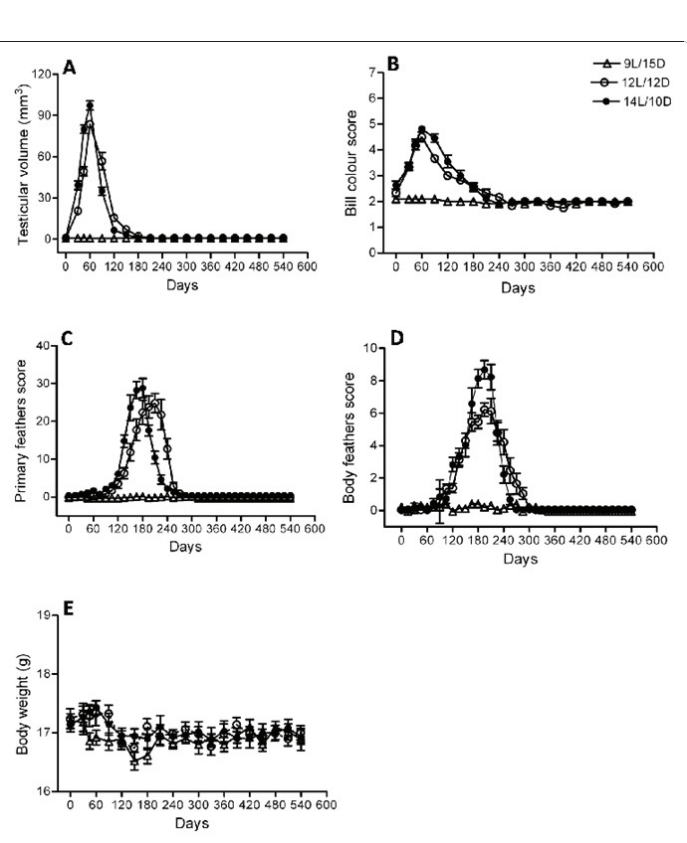
**Effects of constant photoperiods on seasonality in the male tree sparrow (*Passer montanus*)**. Photosensitive birds (n = 35) were exposed to light dark cycles of 9L/15D, 12L/12D and 14L/10D close to shortest, equinox and longest day lengths at Shillong for 540 days with light intensity of ~ 400 lux at perch level in light phage and 0 lux in the dark phage. Food and water were provided *ad libitum. *Periodic changes in testicular volume, bill coloration and body weight were recorded at an interval of 30 days while molt in primary and body feathers were recorded fortnightly during the course of study. Data are presented in means ± s.e.m. Birds showed significant cyclic responses in the above measurements (except in the body weight) only under 12L/12D and 14L/10D while those under 9L/15D failed to do so suggesting the existence of photoperiodic mechanism in their control and exclude the possibility of circannual rhythm generation. Body weight did not appear to be photoperiodically regulated.

Results are presented in Figures 2A, B, C, D and 2E. Sparrows showed significant testicular growth followed by regression and development of photorefractoriness under 12L/12D (F_19, 209 _= 168.9, *P *< 0.0001; one-way RM ANOVA) and 14L/10D (F_19, 190 _= 379.9, *P *< 0.0001) while those exposed to 9L/15D (F_19, 190 _= 1.263, *P *= 0.2123) failed to undergo growth regression cycle in a total duration of 540 days. A significant increase in testicular volume was observed under 14L/10D and 12L/12D on day 30 (*P *< 0.001) and 45 (*P *< 0.001) leading to apparently peak growth on day 60 (*P *< 0.001). Significant testicular regression occurred on day 90 (*P *< 0.001) which progressed rapidly on days 120 and 150 reaching to minimal testicular volume on day 180 under 14L (TV = 0.8 ± 0.1 mm^3^) and on day 210 under 12L (TV = 0.7 ± 0.1 mm^3^) that were maintained till the end of the experiment on day 540. The testicular response cycle was significantly different among the three light regimes (photoperiod F_2, 620 _= 526.5, *P *< 0.0001; duration of exposure F_19, 620 _= 457.5, *P *< 0.0001; photoperiod ↔ duration of exposure F_38,620 _= 126.6, *P *< 0.0001; two-way ANOVA). Though the timing of peak testicular growth was similar under both the stimulatory light regimes, the testicular growth and regression were faster in the birds under 14L/10D as compared to 12L/12D. Change in bill color ran parallel to testicular cycle attaining maximum score on day 60 (mean bill color score; 14L = 4.81 ± 0.12 and 12L = 4.50 ± 0.19) followed by decline on subsequent observations until it reached to minimum score on day 240 (14L = 1.90 ± 0.09) and day 270 (12L = 1.83 ± 0.11) under both 14L/10D (F_19, 190 _= 74.47, *P *< 0.0001) and 12L/12D (F_19, 209 _= 50.37, *P *< 0.0001; one-way RM ANOVA). No significant change in bill color was seen in the birds maintained under 9L/15D (F_19,190 _= 1.230, *P *= 0.2370; Figure 2B). A comparison by two-way ANOVA also indicated significant difference in bill coloration among the three light regimes (photoperiod F_2,620 _= 208.9, *P *< 0.0001; duration F_19,620 _= 102.4, *P *< 0.0001; interaction F_38,620 _= 22.78, *P *< 0.0001). Though no significant different was observed in the increase of bill colour score during its darkening on days 30, 45 and 60 under 14L and 12L, the decrease in bill colour score during its lightning ran faster under 12L on days 90 (P < 0.001) and 120 (P < 0.01) in contrast to testicular regression. Further, the bill colour score reached to its minimum value faster under 14L (day 240) as compared to 12L (day 270) but the differences were found to be statistically insignificant. No significant molt was observed either in the wing primaries or in body feathers of the birds held under 9L/15D (wing primaries F_36,360 _= 0.7172, *P *= 0.8878; body feathers F_36,360 _= 1.251, *P *= 0.1590; one-way RM ANOVA) throughout the course of the experiment. On the other hand, birds showed significant molt under 12L/12D (wing primaries F_36, 396 _= 34.87, *P *< 0.0001; body feather F_36,396 _= 39.28, *P *< 0.0001) and 14L/10D (wing primaries F_36,360 _= 66.36, *P *< 0.0001; body feather F_36,360 _= 58.35, *P *< 0.0001; one-way RM ANOVA). Significant difference in molting response was observed among various light-dark cycles for wing primaries (photoperiod F_2,1147 _= 183.7, *P *< 0.0001; duration of treatment F_36,1147 _= 76.55, *P *< 0.0001 and interaction, F_72,1147 _= 25.35, *P *< 0.0001) and body feathers (photoperiod F_2,1147 _= 212.18, *P *< 0.0001; duration of treatment F_36,1147 _= 86.45, *P *< 0.0001; interaction F_72,1147 _= 22.36, *P *< 0.0001; two-way ANOVA). Further, feather replacement coincided with the testicular regression under gonadostimulatory light regimes. Data on molt score revealed that molt in wing primaries started earlier in 14L/10D as compared to 12L/12D while it progressed simultaneously in case of body feathers. The molt in both types of feathers was completed almost at the same time under 12L and 14 L photoperiods. There was no significant change in the body weight of the birds in all the three light regimes (14L/10D: F_19,190 _= 1.060, *P *= 0.3960; 12L/12D: F_19,209 _= 1.538, *P *= 0.0753; 9L/15D: F_19,190 _= 1.587, *P *= 0.0626; one-way RM ANOVA) throughout the course of experiment.

### Experiment 3: critical day length for testicular response

**Figure 3:**
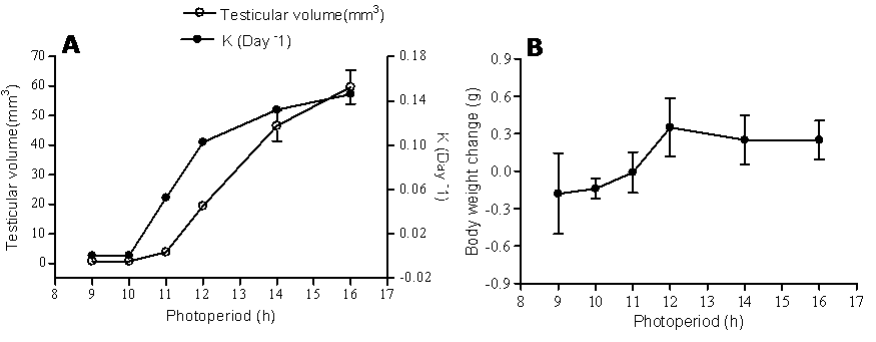
**The critical day length for testicular response in the tree sparrow (*Passer montanus*)**. Photosensitive birds were exposed to six different light regimes (9L/15D, 10L/14D, 11L/13D, 12L/12D, 14L/10D and 16L/8D) at fix intensity of light (L = 400 lux; D = 0 lux) for a period of 30 days. Observations were made on testicular volume and body weight at the beginning and end of the experiment and the testicular growth rates (k) were calculated using the formula; K = (ln_b_-ln_a_)/t where a and b are initial and final testicular volumes, respectively and t is time in days. Significant increase in testicular volume was recorded only when the day length was 11 h/day suggesting that the birds have a critical day length for testicular response above which the rate of growth increases with increasing photoperiods. Birds maintained their normal body weight in all the light regimes showing no significant change.

One way ANOVA exhibited significant variation in testicular volume in the sparrows exposed to various light dark cycles (F_5,54 _= 63.08, *P *< 0.0001; Figure 3A). Birds did not respond to daily photoperiods of 9 and 10 h but significant increase (P < 0.0001, t-test) in testicular volume was observed under 11L/13D, 12L/12D, 14L/10D and 16L/8D. The rate of testicular growth was observed to be greater under longer photoperiods. The mean testicular volume in the birds under 12L/12D or 14L/10D or 16L/8D was significantly higher than in the birds subjected to 11L/13D (*P *< 0.0001, t-test). The mean testicular volume of the birds was significantly more in 14L/10D (P = 0.0003, t-test) or 16L/8D (P < 0.0001, t-test) when compared to birds in 12L/12D. Further, there was significant difference between the mean testicular volumes of the birds under 14L/10D and 16L/8D (P = 0.0180, t-test). Differences in body weight under different light regimes were found to be insignificant (Figure 3B: F_5,54 _= 1.205, *P *= 0.3191; one-way ANOVA).

## Discussion

It is evident from Figures 1 and 2 that the tree sparrows of the study population are photoperiodic and absolutely photorefractory. The annual testicular cycle of wild tree sparrows follows the annual solar cycle at Shillong remarkably well (Figures 1A, B and 1C). The testicular growth was triggered by increasing day lengths of spring when the photoperiod was less than 12 h per day. The birds showed a slight increase in testicular volume in February and March but a significant testicular development was observed only in April and May (P < 0.001, one-way ANOVA). The gonads regressed in summer month (June) when the day lengths were still longer than the spring months indicating the onset of photorefractoriness. This post reproductive refractory period in the tree sparrow is very much similar to what has been described in the annual reproductive cycles of many bird species [39-42]. The annual reproductive cycle of tree sparrow can be divided into four distinct phases with single annual reproductive peak, i.e., preparatory (December-January), progressive (February-March), reproductive (April-May) and regressive (June-November) phases. The annual testicular and molt cycles of tree sparrow (Figures 1C and 1D) at 25°N, 91°E are comparable to those of its conspecifics and other closely related species living at lower and higher latitudes. Molting in the wing primaries and body feathers progressed with gonadal regression extending from June to November with peaks in the month of August in the study birds. These responses are more or less similar to those of sparrow populations living at other latitudes. A study on tree sparrow in Singapore (1°N) reported that it breeds mainly from January to mid-May. There is no overlap of the breeding season with the molt of primary feathers which terminates by late August when the testes is fully regressed. Further, in Malaysia (3°N), they possess significantly large gonads in month of December that lasts for seven to eight months (June-July). Body molt was observed throughout the year except in the month of May with a high proportion in August to November. Similarly, molt in the primary feathers was observed from August to October followed by three months (November-January) when there was no molt in primaries [43]. Thus, the tree sparrows at 1°N and 3°N show a much longer breeding season as compared to their population in Shillong (25°N). On the other hand, population at the higher latitude Poland (52°N) showed a breeding season extending from mid-April to early-August [44]. The above studies on different populations of tree sparrows at various latitudes indicate that they breed later and for a shorter duration with increasing latitudes with the exception of Malaysian population at 3°N. Birds at this latitude had undeveloped testes in November and their testes began to develop in December and into January, and young were observed in February. By contrast, tree sparrows at other latitudes do consistently not begin to breed until March-April. It is important to note here that enriched commercial poultry feed was freely available to Malaysian birds throughout the study while seasonally abundant natural food was available to the birds in other studies. Molting pattern and molt breeding overlap in Malaysian tree sparrows indicate that the increased food availability is the main factor which prolongs breeding season and overlaps two high energy demanding processes of reproduction and molt. The curtailment of breeding and molt in the Malaysian tree sparrow population, in spite of continuous and unlimited availability of high quality food, indicates that the occurrence of these activities can be extended under favourable food condition up to a point. The ultimate extent of breeding may alternatively be limited by the availability of the suitable food to feed the young, and by the necessity to molt, which appears to be regulated by endogenous schedule to avoid the heavy rain [43]. Photoperiod in this case may have a limited role. However, more information based on long term studies on Malaysian population depending only on naturally occurring foods is required to ascertain the role of photoperiod and/or endogenous rhythm and their interaction with food in control of breeding activities.

A comparison of reproductive and associated cycles between our birds at Shillong and the house sparrow, a related species, revealed that the subtropical population of house sparrow at 27°N showed largest gonad in April but the gonads were fully regressed in July. Thus, the active period of gonadal function was longer in tree sparrows than in the house sparrows in the subtropics. Molt began in the same month (June) and completed two months earlier in September in house sparrow [39]. Another study on house sparrows at 52°N reported peak testicular development in May, similar to tree sparrow but full testicular regression was observed one month earlier in August [40]. Thus, tree sparrow at 25°N show photoperiodic responses similar to many related species at higher latitudes. The African stonechats (*Saxicola torquata axillaris*), when subjected to day length variations with an amplitude of 7 h simulating with those occurring at 47.58°N, behaved essentially like temperate zone stonechats (*S. t. rubecula*) in respect of synchronisation of gonadal and molt cycles to either twelve or six months suggesting the conservation of photoperiodic response in them which is independent of the subspecies' origin or present migratory status. However, both the subspecies failed to synchronize their rhythms to six months photoperiodic cycle with amplitude of 1 h and 10 mins. Most temperate zone birds maintained their enlarged testis for six to eleven months while testis started to regress within only two to three months in equatorial birds. The above difference probably reflects evolutionary adjustment of the two subspecies to different photoperiodic conditions prevailing in their respectively breeding ground. The equatorial birds initiate refractoriness under shorter photoperiods than do the temperate-zone stonechats [45]. Further, the equatorial andean sparrow (*Z. capensis*) show a limited capacity for adaptive response to photoperiodic conditions outside the tropics. Unlike its north-temperate relatives (*Z. leucophrys, Z. atricapilla, and Z. albicollis*), the andean sparrow has no photoperiodic response mechanism for effectively preventing late summer and autumn breeding under northern seasonal conditions [46]. The timing of breeding season in the great tits in Netherlands remained unaffected by increasing spring temperature even though selection for earlier breeding was intensified due to earlier highest caterpillar abundance [47]. This may be due to their rigid photoperiodic control system that prevents an adaptive advancement of breeding season. In general, birds which rely on a single, rigid response mechanism for timing reproduction may be unable to immediately track changes in the seasonal availability of food. Range shifts are caused by changes in the geographical distribution of suitable climatic condition and concomitant shifts in resource availability causing birds to follow the environmental condition they are adapted to and evade areas which are no longer optimal. This is the most probable response of birds to global climatic change besides phenological changes. Latitudinal range shifts involve change in photoperiodic condition to which the birds are bound to respond. If optimal time of breeding advances due to sudden rise in global temperature, adaptation of the timing of breeding will be most severely constrained in northern temperate species with relatively high photoperiodic response thresholds. On the other hand if tropical birds (southern origin) with relatively low thresholds extend their ranges to the north, they will become exposed to steeper vernal increase in the day length, which could result in unseasonably early gonadal development and egg laying. However, if novel photoperiodic condition brings forth changes in annual rhythmicity that are adaptive under altered climatic conditions, adaptation to environmental change may be facilitated, or even reinforced [48].

The results obtained from the experiment 2 and subsequent statistical analysis of the data (Figures 2A, B, C, D and 2E) reveal that the tree sparrow is photosensitive. Its photoperiodic responses resemble to those of many north temperate birds in which long daily photoperiods cause gonadal growth followed by regression and onset of photorefractoriness, whereas short photoperiods, by failing to stimulate hypothalamo-hypophyseal complex, do not [8,17,49]. Although seasonal changes in day length is thought to be too small to serve as a cue for timing seasonal events in the tropics and subtropics [42], some birds show photoperiodic control of their seasonal responses. However, it is still difficult to discern general pattern of photoperiodic responses among the birds of these regions because so few of them have been studied. Some sedentary low latitude forms, including weaver bird (*Ploceus philippinus*) and rufous-collared sparrow (*Zonotrichia capensis*), show gonadal response to long daily photoperiod, but do not become photorefractory [50,51]. However, other sedentary forms behave quite differently. In black-headed munia (*Munia malacca mallaca*), for example, long as well as short day (ranging from 8-24 h) are gonadostimulatory [52]. Spotted munia (*Lonchura punctulata*) responds to unnaturally short photoperiods (ranging from 0.25-6 h) but the customary long and short days (ranging from 8-24 h) fail to stimulate gonadal growth; long days rather retard or inhibit it [53], red-billed quelea (*Quelea quelea*) are photoperiodic exhibiting brief refractory period that terminates spontaneously irrespective of photoperiodic condition [54]. Our study on the photoperiodic responses of tree sparrow stands in contrast to those mentioned above but resembles to those of some subtropical birds, e.g., red-headed bunting (*Emberiza bruniceps*) [55,56], black-headed bunting (*Emberiza melanocephala*) [57], rosefinch (*Carpodacus erythrinus*) [24,58], yellow-throated sparrow (*Gymnorhis xanthocollis*) [59] and house sparrow (*Passer domesticus) *[39]. Though the timing of peak gonadal growth was similar under both the stimulatory light regimes in the tree sparrow, the testicular growth and regression were faster in the birds under 14L/10D as compared to 12L/12D (Figure 2A). A similar response was observed in the house sparrow exposed to 14L, 18L and 22L at 27⁰N [39] and 13L, 16L and 18L photoperiods at 52⁰N [60]. Data presented in figure 2A are in agreement with the reports that longer the photoperiod, faster is the rate of gonadal growth and subsequent regression and narrower is gonadal growth phase [22,61,9].

It has been demonstrated in few bird species that the annual cycles of various physiological and behavioral functions are controlled by an endogenous circannual rhythmicity [62]. Under seasonally constant environmental conditions, these rhythms persist for several cycles with a period usually slightly different from twelve months. Further, it has been suggested that the annual change in day length acts as zeitgeber that entrains an endogenous circannual rhythm [63,64,25]. Schwab [65] has clearly shown that the male starlings (*Sturnus vulgaris*) maintained in the laboratory on a light regime of 12L/12D for about two years show periodic fluctuation in gonadal size that corresponds remarkably to the natural testicular cycle of the wild Starling. Lofts [66] reported similar observation in red-billed quelea under 12L/12D. It seems that the annual cycles of these birds are timed by an endogenous circannual clock that retains its seasonal accuracy by being reset a new by environmental or social stimuli [17]. In contrast, tree sparrow in our study not only failed to respond to short day length (9L/15D) but did not show regrowth of their testes after complete gonadal initiation-regression cycle when maintained under gonadostimulatory photoperiods (12L and 14L) that approach to those that feral birds experience in the nature in a total duration of eighteen months. A long day species usually do not show gonadal response under short photoperiods (light below critical day length) and this indicates the importance of photoperiodic cues over an endogenous circannual rhythm in control of reproductive cycle of tree sparrows. On the other hand, gonadal recrudescence under short day length in a long day breeder may be the consequence of seasonal rhythm rather than of the photoperiod.

In the present study, molt of wing primaries and body feathers progressed with the gonadal regression (Figures.2C and 2D). The initiation of molt has been found to be linked to a decreased reproductive activity in a number of avian species [67-69]. Though a complete molt was observed in the birds under long photoperiods, it began earlier and was more pronounced in 14L than 12L. Since gonadal regression was faster in 14L as compared to 12L, we can infer that faster the gonadal regression earlier is the onset of molt in the tree sparrow. A similar molt pattern under stimulatory photoperiods has been reported in house sparrows at 27⁰N [39], 52⁰N [40] and in many other avian species [70]. On the other hand, birds do not undergo feather replacement under non-gonadostimulatory photoperiod (9L/15D) suggesting that photostimulation is required to induce molt that also induces gonadal growth and then regression in sparrows. It is not clear whether photoperiod has a direct effect on molt, or it is secondary consequence of photoperiodic stimulation of gonadal cycle and a physiological link between gonadal regression and molt, meeting the ecological requirement for molt to immediately follow breeding [4].

The data presented in Figure 3A indicate that light falling for 11 h is important in inducing testicular growth in sparrows as 10 h daily photoperiod failed to induce testicular response while the birds experiencing 11 h light per day responded significantly. Thus, tree sparrow in the present study may have a photoperiodic threshold equal to or even bit less than 11 h. The minimum photoperiod that induces a photoperiodic response appears to be species specific, varying with the duration of experiment and may be adapted for breeding at a particular time of the year, at particular latitude [71,59,8]. It may also vary among migratory and non-migratory forms of the same species [72]. The threshold photoperiods for gonadal growth for some tropical and subtropical birds are reported to lie between 11-12 h in weaver bird [73]; black-headed bunting [57]; red billed dioch [54] and between 12-13 h in red-headed bunting [74]; rose finch [24] and yellow-throated sparrow [59]. They are, thus, close to the photoperiodic requirement of tree sparrow for gonadal growth. The tree sparrows have not only a definite threshold for gonadal sensitivity but also the rate of growth increases with increasing photoperiods (Figure 3A). The steepness of photoresponse curve in the tree sparrow is comparable to those of males of Japanese quail (*Coturnix coturnix japonica*) that show low rate of maturation when the photoperiod is less than 11.5 h of light period per day, somewhat greater on 11.5 h and rapid on 12 h [61]. As the photoperiod increases during the spring, gonadal maturation begins when it reaches this critical threshold. However the concept of critical photoperiod requires a cautious approach. In tree sparrow, the gonadal growth rate changes rapidly over a short range of photoperiods birds experience in the nature, so the definition of critical photoperiod is easy. In starlings [75] and white-crown sparrows (*Zonotrichia leucophrys gambelli*) [76], on the other hand, gonadal growth rate varies over a wider range of photoperiod. Tree sparrows in the wild showed initiation of gonadal growth in February/March that continued up to May (Day length: 11-13.28 h) as predicted from the photoresponse curve (Figure 3A). Thus, the natural time of gonadal growth in the sparrow corresponds to a photoperiodic threshold under laboratory conditions. These observations further support our assumption that, despite the small annual variation in day length of tropics and subtropics (3 h and 15 minutes at Shillong, India; 25°34'N, 91°53'E) the tree sparrow might be using increasing photoperiods of spring as a cue in regulating its reproductive seasonality in nature. This is supported by a study in Central Panama (9°N) that a small increase in daylength of only 17 minutes was sufficient to induced gonadal growth in spotted antbird (*Hylophylax n. naevioides*) which experience annual variation in photoperiod of only 1 h in the nature [21]. Since tree sparrows share their habitat with yellow-breasted bunting (*Emberiza aureola*) at this latitude, they are expected to have similar photoperiodic mechanisms. As expected, both use increasing day length of spring to initiate their gonadal development cycle (our unpublished data).

In our study, no significant change in body weight was observed in wild as well as the light treated birds under different experiments and no significant deposition of fat was observed at any stage (Figures 1D, 2E and 3B). These results clearly follow the suggestions that fattening is generally lacking in non-migratory birds and if fattening occurs, it is only to a limited extend [16] that accounts for minor but insignificant changes in the body weight in the tree sparrows. Since the fat serves the purpose of stored food and fuel during migratory flights, its deposition in tree sparrow (a non-migratory resident bird) that has easy access to food in the surrounding, would hamper its flight activity. Bill coloration, in tree sparrows, runs almost parallel to testicular cycle attaining peak in the months of April and May suggesting the possibility of its control by increasing gonadal steroids from photostimulated gonad. Similar observation was recorded in subtropical red-headed bunting [77]. There are several lines of evidence suggesting that bill color is a testosterone dependent trait in some birds including zebra finch (*T. guttata) *[78] and Eastern American goldfinch (*Spinus t. tristis) *[79]. However, further investigation is required to ascertain the role of gonadal steroids in control of bill colour in the tree sparrow as bill colour lightened faster under 12L (days 90 and 120) while the testis regressed faster under 14L (days 90 and 120), though the bill colour score reached to its minimum value faster under14L (day 240) as compared to 12L (day 270).

## Conclusion

In conclusion, our study provide evidence that the subtropical tree sparrow is a photosensitive bird that can detect changes in the length of daily photoperiods as small as those normally experienced by the birds in Shillong. In spite of significantly different environmental conditions at 25⁰N, 91⁰E, this bird shows photoperiodic responses similar to those of its populations and closely related species living at high latitudes, e.g. 27⁰N and 52⁰N. This suggests that the tree sparrow at relatively low latitude continues to utilize annual solar cycle as a proximate environmental cue to time its seasonal responses. Thus, the present study on tree sparrow shows conservation of photoperiodic control mechanisms as an adaptive strategy in temporal environment ensuring seasonal events to occur at the most suitable time of the year.

## Methods

### Capture, maintenance and pretreatment

Adult male tree sparrows (*Passer montanus *Linneus), a non-migratory resident species, were captured in and around the hills of Shillong (Latitude 25°34"N, Longitude 91°53"E) in the fall of 2008 and kept in an outdoor aviary. This aviary is located in the vicinity of our department in an open area surrounded by natural vegetation and receiving natural light and temperature conditions. Testes at this time were completely regressed and the birds had no conspicuous subcutaneous fat. These birds were then acclimatized to laboratory conditions for a fortnight. There they were subjected to natural variations of photoperiod, temperature and humidity. The birds were then transferred to the short day length (9L/15D) for eight weeks to eliminate photorefractoriness if they had any in nature and to ensure their photosensitivity at the time of commencement of various experiments. Laparotomy (surgical opening of abdominal wall between the last two ribs) at four weeks intervals during the pretreatment period revealed that they had regressed testis. These photosensitive birds were used in different photoperiodic investigations. Study of annual seasonal cycles was made on the wild birds captured in the middle of every month of the year 2009. Three experiments were performed using adult male tree sparrow.

### Experimental design

#### Experiment 1: seasonal cycles in the wild birds

This experiment involved the study of seasonal cycles in relation to annual variation in various environmental factors at Shillong. Birds (n = 7) were captured from the wild in the middle of every month of the year 2009 and observations were recorded on testicular volume, molting pattern, bill coloration and body weight.

#### Experiment 2: seasonal cycles under programmed schedules

The second experiment was performed in order to know whether photoperiod has a role in timing seasonal cycles in this bird. The experiment began in January 2009 and continued up to June 2010 for 540 days. Photosensitive birds (n = 35) were kept under three different photoperiods i.e., 9L/15D, 12L/12D and 14L/10D close to shortest, equinox and longest day lengths, respectively at Shillong. Data on body and primary flight feathers were recorded fortnightly and on testicular volume, bill coloration and body weight at an interval of 30 days during the course of study.

#### Experiment 3: critical day length for testicular response

In this experiment, a combination of light and dark in 24 h cycle with increasing proportion of light period was used to find out minimum photoperiod required to induce testicular growth. Birds were kept under increasing photoperiods, i.e., 9L/15D, 10L/14D, 11L/13D, 12L/12D, 14L/10D and 16L/8D for 30 days and testicular volume and body weight were recorded at the beginning and end of the experiment.

### Experimental conditions and measurements

Birds, in photoperiodic experiments, were kept in light proof wooden chambers (size- 7 × 4 × 4^1/2 ^ft.) illuminated by light available from compact fluorescent tubes (CFL, Phillips) at an intensity of ~ 400 lux at the perch level. Light on and off were controlled by automatic digital time switches (Crono Digital Time Switches, Larsen and Toubro LTD., India). Our photoperiodic chambers are well aerated through inlets and outlets connected to air circulators. The temperature and humidity of experimental chambers, as recorded by HOBO data logger, varied in the ranges of about 17°C (December) - 24°C (June) and 55-75%, respectively in a year. Food and water were available *ad libitum *and were replenished only during the light phase of the cycle. The testicular size was recorded by performing laparotomy under local anaesthesia using subcutaneous injection of 2% xylocaine (Astra-IDL Ltd. Bangalore, India) as per the procedure described in Kumar et al. [80]. Briefly, laparotomy was performed by surgical opening of the abdominal wall between the last two ribs on the left side, testis was located within the abdominal cavity with the help of a spatula and the length and width of the left testis was measured. Testis volume was calculated using formula 4/3π*ab*^*2*^, where *a *and *b *denote half of the long (length) and short (width) axes, respectively. The molt pattern was recorded by observations on primary wing feathers (called primaries) and body feathers. The testicular growth rate (k) was calculated using the formula; K = (ln_b_-ln_a_)/t where a and b are initial and final testicular volume, respectively and t is time in days. For primaries, we followed scoring pattern as outlined by Boswell [81] in a scale of 0-5 as per the following: 0 = worn or old feather, 1 = missing feather (just dropped), 2 = from a new feather papilla emerging up to one-third growth, 3 = new feather attaining two-third growth, 4 = new feather grown, but still the growth is not fully complete, 5 = fully grown new feather. Thus, each primary feather can posses a minimum score of 0 and maximum of 5. As there are nine primaries on each wing, the maximum score per wing could be up to 45 (9 × 5 = 45), and for each bird a maximum score of 90 (2 × 45) could be expected. Minimum score could be as low as 0. For recording of body molt, whole body of the bird was divided into 12 different regions: 1 = head, 2 = neck, 3 = shoulder, 4 = back, 5 = pelvic, 6 = caudal, 7 = throat, 8 = chest, 9 = abdomen, 10 = flank, 11 = shank and 12 = sub-caudal as described by Puja et al. [82]. Each region could have a score of either 0 (no molt: fully grown or old feather present) or 1 (molt: no feather present or new feathers emerging) and hence the total body molt score could be in the range of 0-12. The bill color was scored in an index of 0-5: 0-Bill straw in color (S), 1-Bill straw in color but with a little tinge of blackness (ratio-SSS:B), 2-Bill slightly blackish in color (ratio-SS:B), 3-Bill straw and black in approximately 50:50 patches (ratio-S:B), 4-Bill black with very little straw patch left (ratio-S:BB), 5-Bill completely black (B) as mentioned in Malik et al. [83]. Body weight was measured using a top pan balance to an accuracy of 0.1 g.

The data from different experiments are presented as mean ± s.e.m. They were analyzed using one-way analysis of variance with repeated measures (one-way RM ANOVA), as required, followed by Newman-Keul's Multiple range't' test if ANOVA indicated a significance of difference. Two-way ANOVA was used to compare when two factors (e.g. photoperiod and duration) were involved in the experiment. Significance was taken at *P *< 0.05. Also, students't' test was used while comparing only two means.

## Competing interests

The authors declare that they have no competing interests.

## Authors' contributions

ASD conceived of the study, participated in its design and coordination, analysed the data and drafted the manuscript. NSS carried out photoperiodic experiments and performed statistical analysis. All authors read and approved the final manuscript.
